# Hospital Notes

**Published:** 1896-02

**Authors:** 


### Hospital Notes.

The Children's Hospital, of Bryant street, Buffalo, was liberally remembered during the holiday season. On New Year's day the sum of $5,000 was received from J. N. Adam for the endowment of a free bed, in memory of his wife, Margaret L. P. Adam. The same day a check for $3,000 was received from Mr. and Mrs. Clarence O. Howard for the endowment of a bed as a memorial to their little daughter Josephine. During the month of December the board received the sum of $3,000 from Mr. and Mrs. George H. Lewis for a free bed in memory of their little son Freddie. The Children's Hospital is now in possession of four endowed beds, which means vast benefit to hosts of helpless children. It is announced that Miss Martha Williams, who has been a staunch friend and liberal supporter of the hospital from the outset, will build an annex on the hospital lot, to be called the Folwell memorial pavilion in memory of the late Dr. Folwell, which will provide increased accommodations for the now overflowing wards.

---

The Erie County Hospital medical staff held its annual meeting at the Buffalo Academy of Medicine, Monday evening, January 6, 1896. The following officers were unanimously reëlected for 1896 : President, Dr. J. H. Pryor ; first vice-president, Dr. William S. Tremaine ; second vice-president, Dr. F. Park Lewis ; secretary, Dr. Francis Metcalfe.

The following resolution was presented by Drs. Rochester, Lewis and Howell and was unanimously adopted :

We, the members of the medical staff of the Erie County Hospital, in recognition of the self-sacrificing and effective service of the president of the staff, Dr. John H. Pryor, and in evidence of our appreciation of the great work which he has done in advancing that institution to its present condition, herewith extend to him our sincere thanks and assure him of our hearty coöperation in continuing the work so admirably begun.

